# One simple claudication question as first step in Peripheral Arterial Disease (PAD) screening: A meta-analysis of the association with reduced Ankle Brachial Index (ABI) in 27,945 subjects

**DOI:** 10.1371/journal.pone.0224608

**Published:** 2019-11-04

**Authors:** Arne Georg Kieback, Christine Espinola-Klein, Claudia Lamina, Susanne Moebus, Daniel Tiller, Roberto Lorbeer, Andreas Schulz, Christa Meisinger, Daniel Medenwald, Raimund Erbel, Alexander Kluttig, Philipp S. Wild, Florian Kronenberg, Knut Kröger, Till Ittermann, Marcus Dörr

**Affiliations:** 1 Kantonsspital Aarau- Medizinische Universitätsklinik, Angiologie, Aarau, Switzerland; 2 University Medicine Mainz—Department of Angiology, Mainz, Germany; 3 Medical University of Innsbruck—Institute of Genetic Epidemiology, Department of Genetics and Pharmacology, Innsbruck, Austria; 4 Universitätsklinikum Essen (AöR), Institut für Medizinische Informatik, Biometrie und Epidemiologie (IMIBE), Essen, Germany; 5 Martin-Luther-University Halle-Wittenberg—Institute of Medical Epidemiology, Biostatistics and Informatics, Halle (Saale), Germany; 6 Ludwig Maximilian University Hospital—Department of Clinical Radiology, Munich, Germany; 7 University Medical Center of the Johannes Gutenberg-University Mainz, Center for Cardiology I, Mainz, Germany; 8 Helmholtz Zentrum München—German Research Center for Environmental Health (GmbH)—Institute of Epidemiology II, Neuherberg, Germany; 9 Chair of Epidemiology, Ludwig-Maximilians-Universität München, UNIKA-T Augsburg Augsburg, Germany; 10 University Medical Center of the Johannes Gutenberg-University Mainz, Preventive Cardiology and Preventive Medicine, Center for Cardiology, Mainz, Germany; 11 University Medical Center of the Johannes Gutenberg-University Mainz, Center for Thrombosis and Hemostasis (CTH), Mainz, Germany; 12 DZHK (German Center for Cardiovascular Research), partner site RhineMain, Mainz, Germany; 13 Helios Klinikum Krefeld—Klinik für Gefäßmedizin, Krefeld, Germany; 14 University Medicine Greifswald, Institute for Community Medicine, Greifswald, Germany; 15 DZHK (German Centre for Cardiovascular Research), partner site Greifswald, Greifswald, Germany; 16 University Medicine Greifswald, Department of Internal Medicine B, Greifswald, Germany; NIHR Leicester Biomedical Research Centre, UNITED KINGDOM

## Abstract

**Purpose and methods:**

A meta-analysis using data from seven German population-based cohorts was performed by the German Epidemiological consortium of Peripheral Arterial Disease (GEPArD) to investigate whether one question about claudication is more efficient for PAD screening than established questionnaires. Claudication was defined on the basis of the answer to one question asking for pain in the leg during normal walking. This simple question was compared with established questionnaires, including the Edinburgh questionnaire. The associations of claudication with continuous ABI values and decreased ABI were analyzed by linear and logistic regression analysis, respectively. The results of the studies were pooled in a random effect meta-analysis, which included data from 27,945 individuals (14,052 women, age range 20–84 years).

**Results:**

Meta-analysis revealed a significant negative association between claudication and ABI, which was stronger in men (β = -0.07; 95%CI -0.10, -0.04) than in women (β = -0.02; 95%CI -0.02, -0.01). Likewise, the presence of claudication symptoms was related to an increased odds of a decreased ABI in both men (Odds ratio = 5.40; 95%CI 4.20, 6.96) and women (Odds ratio = 1.99; 95%CI 1.58, 2.51).

**Conclusions:**

Asking only one question about claudication was able to identify many individuals with a high likelihood of a reduced ABI with markedly higher sensitivity and only slightly reduced specificity compared to more complex questionnaires. At least in men, this question should be established as first screening step.

## Introduction

### Peripheral arterial disease

Peripheral arterial disease (PAD) is an underdiagnosed disease with high cardiovascular and cerebrovascular morbidity and mortality[1, 2]. It affects over 200 million adults around the world[3] and is associated with a relevant number of major adverse limb events (MALE)[4, 5]. Therefore current guidelines recommend to implement and support medical and public awareness of PAD[1]. While prognostic improvement of interventional or surgical treatment of stable PAD could not be demonstrated so far, exercise, smoking cessation, healthy diet, weight loss in overweight people and pharmacological prophylaxis may prevent major adverse cardiovascular events (MACE) and major adverse limb events (MALE), especially if PAD is diagnosed at an earlier clinical stage. For example, aggressive lipid lowering therapy can reduce major adverse cardiovascular and limb events in PAD patients[6], with a larger risk reduction in comparison to atherosclerotic patients without PAD.

In a study with 18500 French general practitioners, intermittent claudication was well known, but knowledge of recommendations for further diagnostic workup and treatment of PAD was poor[7].

In order to diagnose most individuals with PAD, regular measurements of the ankle-brachial index (ABI) in the whole population starting at an age of about 40 years seem to be useful. Currently, general practitioners usually do not screen for PAD. ABI measurements need training, equipment and are time- consuming. No established simple and fast screening strategies were available.

While PAD is sufficiently prevalent for screening, increases all-cause and cardiovascular mortality by 2–3 fold and an accurate test (ABI measurement) is available, adequately powered studies on morbidity and mortality reduction and cost-effectiveness of screening have been lacking[8]. However, in the VIVA study, a combined screening approach for PAD (ABI < 0.90 or > 1.4), abdominal aortic aneurysm and hypertension screening versus no screening was tested in 50156 Danish men at age 65–74 years[9]. Screened men, in which one of the above mentioned diseases were found, received pharmacologic treatment (in PAD, daily doses of 75 mg aspirin and 40 mg simvastatin) along with instructions on diet, smoking cessation and exercise. In the screening group, a 7% lower mortality was achieved after a median follow-up of 4.4 years at a cost of € 2148 per gained quality adjusted life year. This result therefore challenges the current guideline recommendation not to prescribe aspirin in patients with asymptomatic PAD[1].

### Ankle-brachial index

The most important criterion for the definition of peripheral arterial disease (PAD) is an ankle-brachial index (ABI) below 0.90. Low ABI is associated with increased mortality and morbidity[10]. Thereby, ABI is not only a diagnostic, but also an important prognostic marker[11]. In clinical routine, ABI measurements are rarely performed, unless claudication symptoms have been mentioned by the patient. These measurements are usually only performed by vascular specialists for diagnosis of PAD and follow-up after interventional or surgical PAD treatment. Use of ABI measurements is also hampered by lack of adequate financial compensation in most areas of Germany.

### Claudication

Claudication as a major clinical symptom of PAD could also be identified as an independent predictor of increased mortality based on data from the population-based Study of Health in Pomerania (SHIP)[12]. Of note, in this study claudication was defined on the basis of a positive answer to only one question asking for pain or cramps in the leg(s) during normal walking for the main analysis. Traditionally, in population-based or large clinical studies, different questionnaires were used to define claudication. Often, the Rose Questionnaire[13–17], Edinburgh Questionnaire[18] or Framingham Questionnaire[19–21] were used. Development of the Rose questionnaire, which has widely been used since its first publication in 1962[22], was based on a small number of patients. While being highly specific (90–100%) in comparison to the diagnosis of intermittent claudication made by a physician, it is not very sensitive (60–68%)[23]. The Edinburgh questionnaire, a simplified modification of the Rose questionnaire, improves sensitivity (91%) and specificity (99%), which was likewise evaluated in comparison to the clinical evaluation of a physician[23] (4). Using questionnaires enables the identification of some symptomatic patients, but is time-consuming and currently not established in primary medical care. However, one question asking for pain in the leg(s) during normal walking could easily be integrated in the ascertainment of a routine medical history.

### Association between claudication and ABI

Large studies analyzing the association between claudication and ABI are missing. Therefore, we performed a meta-analysis within the German Epidemiological Peripheral Arterial Disease (GEPArD) consortium using cross-sectional data from seven population-based cohort studies to investigate the association between claudication and ABI in both sexes, using different definitions of claudication, ranging from one single question to the Edinburgh Questionnaire. Data from seven well-established cohort studies, which cover different regions of Germany, were analyzed.

This analysis was performed, because PAD awareness would be significantly improved if many patients could be identified by a single question of a general practitioner. As individuals with severe PAD are more likely to actively seek physician’s advice, it would especially be of great help to identify those with mild PAD.

## Methods

### The GEPArD consortium and study population

This consortium was founded to jointly perform analyzes on PAD-related research questions using epidemiological data from different German regions. For the current analyses we included data from all recent German population-based cohorts with data on claudication and ABI, we are aware of. Cross-sectional data from seven population-based cohorts were available: Cardiovascular disease, living and ageing in Halle (CARLA)[24], Gutenberg Health Study (GHS)[25, 26], Heinz-Nixdorf-Recall-Studie (HNR)[27, 28], Cooperative Health Research in the Region of Augsburg (KORA) F3, KORA F4[29, 30], and the Study of Health in Pomerania (SHIP)-2, SHIP-TREND[31]. The studies comply with the Declaration of Helsinki, the locally appointed ethics committees have approved the research protocols (CARLA: Ethics Committee of the Medical Faculty of the Martin- Luther- University Halle- Wittenberg; GHS: Ethics committee of the State Medical Council of Rhineland-Palatinate; HNR: Ethics Committee of the Essen University Hospital; KORA: Ethics committee of the Bavarian Chamber of Physicians; SHIP: Ethics Committee of the University of Greifswald) and informed consent has been obtained from all participants. For this meta-analysis anonymized data from participants of 7 population-based cohorts were analyzed.

### Inclusion and exclusion criteria

The above mentioned seven population-based cohorts included men and women who were randomly selected from regional population registries, if they followed the invitation to take part. For this analysis, all participants of these studies with valid data on claudication, ABI and covariates were included. Participants with prior percutaneous transluminal angioplasty or bypass surgery of peripheral arteries, who were already diagnosed with PAD, were excluded from this analysis.

### Assessment of claudication, ABI and covariates

Ankle and brachial blood pressure values were obtained from the first examination of each participating study. In all studies, the highest ankle pressure of each leg was documented and then the leg with the lower ankle pressure was used for the calculations of this analysis. An ABI < 0.90 was defined as low ABI. Claudication was analyzed according to different questionnaires or a single question (Table 1). The association between claudication and ABI was analyzed for three different claudication definitions. Definition 1 used just one question on pain or cramps in the legs while walking. Definition 3 used the questions of the Edinburgh questionnaire (in GHS participants were asked if they experience pain in the calf/calves instead of drawing pain localization in the calf/calves), in definition 2 the question on pain localization in the calf was left out.

**Table 1:** Claudication questions.

Question #	Definition 1	Definition 2	Definition 3 (Edinburgh questionnaire)
1	Do you get a pain in either leg on walking? (Yes, No)	Do you get a pain or discomfort in your leg(s) when you walk? (Yes, No, I am unable to walk)	Do you get a pain or discomfort in your leg(s) when you walk? (Yes, No, I am unable to walk)
2		Does this pain ever begin when you are standing still or sitting? (Yes, No)	Does this pain ever begin when you are standing still or sitting? (Yes, No)
3			Alternative 1 (from Rose questionnaire): Do you get this pain in your calf (or calves)? (Yes, No) Alternative 2 (from pain localization drawing of the Edinburgh questionnaire): „yes”if pain is localized in calf/calves
4		Do you get it if you walk uphill or hurry? (Yes, No)	Do you get it if you walk uphill or hurry? (Yes, No)
5		Do you get it when you walk at an ordinary pace on the level? (Yes, No)	Do you get it when you walk at an ordinary pace on the level? (Yes, No)
6		What happens to it if you stand still? (Usually continues more than 10 minutes, Usually disappears in 10 minutes or less)	What happens to it if you stand still? (Usually continues more than 10 minutes, Usually disappears in 10 minutes or less)
**Analyzed in:**	**All studies**	**CARLA, GHS, KORA F3, KORA F4**	**GHS, KORA F3, KORA F4**

Information on covariates was derived from interviews or by standardized measurements. Smoking status was classified as never/ex-smokers or current smokers. Hypertension was defined by either self-reported use of antihypertensive medication or a systolic BP ≥140 mmHg and/or a diastolic value ≥ 90 mmHg (calculated by mean of the second and third value out of three measurements, assessed after a 5 min resting period in sitting position). Diabetes mellitus was defined by self-reported physician’s diagnosis or use of antidiabetic medication (ATC code A10) or by HbA1c≥6.5%. Use of lipid modifying medication was assessed based on ATC coding (code C10). Waist circumference (WC) was assessed to the nearest 0.1 cm using an inelastic tape measure. Serum levels of low density lipoprotein (LDL) and high density lipoprotein (HDL) cholesterol were assessed by the methods described for each cohort previously.

### Statistical analyses

The association between claudication with continuous ABI values was analyzed in a linear regression analysis for each cohort separately. Likewise, the association between claudication with low ABI values was analyzed in a logistic regression analysis on cohort-level. The results of the population-based studies were then pooled in a meta-analysis. Study-specific effect sizes for the association between claudication and ABI were combined to a pooled effect size by random-effect meta-analysis[32]. Random effects were applied because the I^2^ pointed towards heterogeneity of the estimates across the studies assuming that the true effect sizes are significantly different between the studies as indicated by the p-value for heterogeneity provided in the graphics. Meta-analysis was conducted using the metan command in STATA 13.1 (Stata Corporation, College Station, TX, USA). Based on data from the largest cohort, GHS, additionally sensitivity and specificity of each of the three definitions to detect low ABI was calculated for two different age-groups (≥55 to <65 years, ≥65 to <75 years). The methods were carried out in accordance with the relevant guidelines and regulations.

## Results

Overall data of 27,945 subjects (14,052 women, age range 20–84 years) could be included into the respective analyses. Characteristics of the samples are shown in Table 2.

**Table 2:** Characteristics of study samples.

Characteristic	CARLA	KORA F3	KORA F4	GHS	HNR	SHIP-2	SHIP-TREND
**N**	1779	2901*	1730*	14342	3252	1386	2555
**Age, years**	64 (55; 73)	57 (46; 67)	64 (58; 71)	55 (46; 64)	59 (53; 66)	57 (46; 67)	53 (41; 64)
**Men, n (%)**	967 (54 %)	1413 (48.7%)	857 (49.5%)	7165 (50.0%)	1535 (47.2%)	686 (49%)	1270 (50%)
**Hypertension, n (%)**	1405 (79 %)	1439 (49.6%)	902 (52.1%)	7094 (49.5%)	1863 (57.3%)	731 (53%)	1200 (47%)
**Diabetes mellitus, n (%)**	274 (15 %)	226 (7.8%)	178 (10.3%)	1305 (9.1%)	377 (11.6%)	184 (13%)	275 (11%)
Family History of MI, n (%)**	105 (6%)	75 (2.6%)	79 (4.6%)	2355 (16.4%)	n.a.	48 (3.5%)	73 (2.9%)
Family History of stroke, n (%)**	69 (4%)	63 (2.2%)	49 (2.8%)	1278 (8.9%)	n.a.	38 (2.8%)	45 (1.8%)
**Smoking status Current smoker, n (%) Ex-smoker, n (%) Never smoker, n (%)**	344 (19 %) 636 (36%) 798 (45%)	546 (18.8%) 1068 (36.8%) 1287 (44.4%)	213 (12.3%) 714 (41.3%) 803 (46.4%)	2775 (19.3%) 4970 (34.7%) 6597 (46.0%)	811 (24.9%) 1120 (34.4%) 1321 (40.6%)	248 (18%) 606 (44%) 532 (38%)	588 (23% 982 (38%) 985 (39%)
**Total cholesterol, mmol/L**	5.44 (4.81; 6.13)	5.58 (4.94; 6.26)	5.72 (5.07; 6.41)	5.67 (4.97; 6.37)	5.93 (5.25; 6.57)	5.40 (4.70; 6.20)	5.40 (4.70; 6.20)
**HDL cholesterol, mmol/L**	1.33 (1.10; 1.63)	1.45 (1.19; 1.76)	1.40 (1.17; 1.68)	1.42 (1.19; 1.74)	1.50 (1.19; 1.78)	1.40 (1.16; 1.67)	1.40 (1.17; 1.66)
**LDL cholesterol, mmol/L**	3.22 (2.65; 3.83)	3.28 (2.71; 3.83)	3.59 (3.00; 4.22)	3.55 (2.95; 4.17)	3.77 (3.13; 4.37)	3.33 (2.70; 3.97)	3.35 (2.72; 3.98)
**Lipid lowering drugs, n (%)**	295 (17 %)	313 (10.8%)	327 (18.9%)	1881 (13.1%)	434 (13.4%)	260 (19%)	353 (14%)
**Waist circumference, cm**	100 (92; 108)	95 (85.4, 103.5)	96.55 (88.40; 105.1)	93.6 (84.7; 103.1)	103.0 (96.3;108.2)	92 (82; 101)	90 (80; 100)
**Claudication Definition 1 Definition 2 Definition 3**	552 (31 %) 36 (2 %) 36 (2 %)	600 (20.7%) 161 (5.5%) 75 (2.6%)	280 (16.2% 74 (4.3%) 53 (3.1%)	1091 (7.6%) 284 (2.0%) 201 (1.4%)	185 (5.7%)—-	130 (9.4)—-	194 (7.6%)—-
**Mean ABI**	1.19 (1.12; 1.26)	1.12 (1.05; 1.20)	1.16 (1.08; 1.23)	1.04 (0.99; 1.10)	1.10 (1.03; 1.18)	1.12 (1.07; 1.18)	1.12 (1.07; 1.18)
**Decreased ABI (<0.9)**	77 (4.4 %)	108 (3.7%)	96 (5.5%)	763 (5.3%)	202 (6.2%)	33 (2.4%)	31 (1.2%)

### Frequencies of claudication and ABI values

The proportion of participants complaining of claudication varies largely between the different cohorts with a minimum of 5.7% (HNR) and a maximum of 31% (CARLA) when definition 1 (asking one question for pain in the leg/ legs one walking) (Table 2) was used. According to the definitions 2 and 3 (Table 2), the proportion of affected subjects is markedly lower with much smaller inter-cohort differences (between 1.4% in GHS for definition 3 and 5.5% in KORA F4 for definition 2).

The unadjusted mean ABI reached from 1.04 to 1.19 among the participating studies, while the proportion of participants with low ABI varied between 1.2% and 6.2% (Table 1).

### Association between claudication and ABI

**Fig 1:**
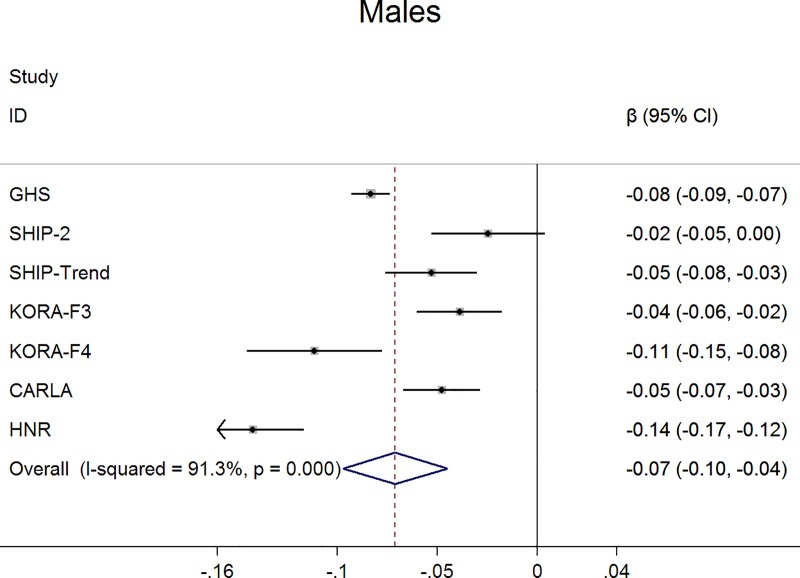
Association between claudication (definition 1) and ABI in men.

**Fig 2:**
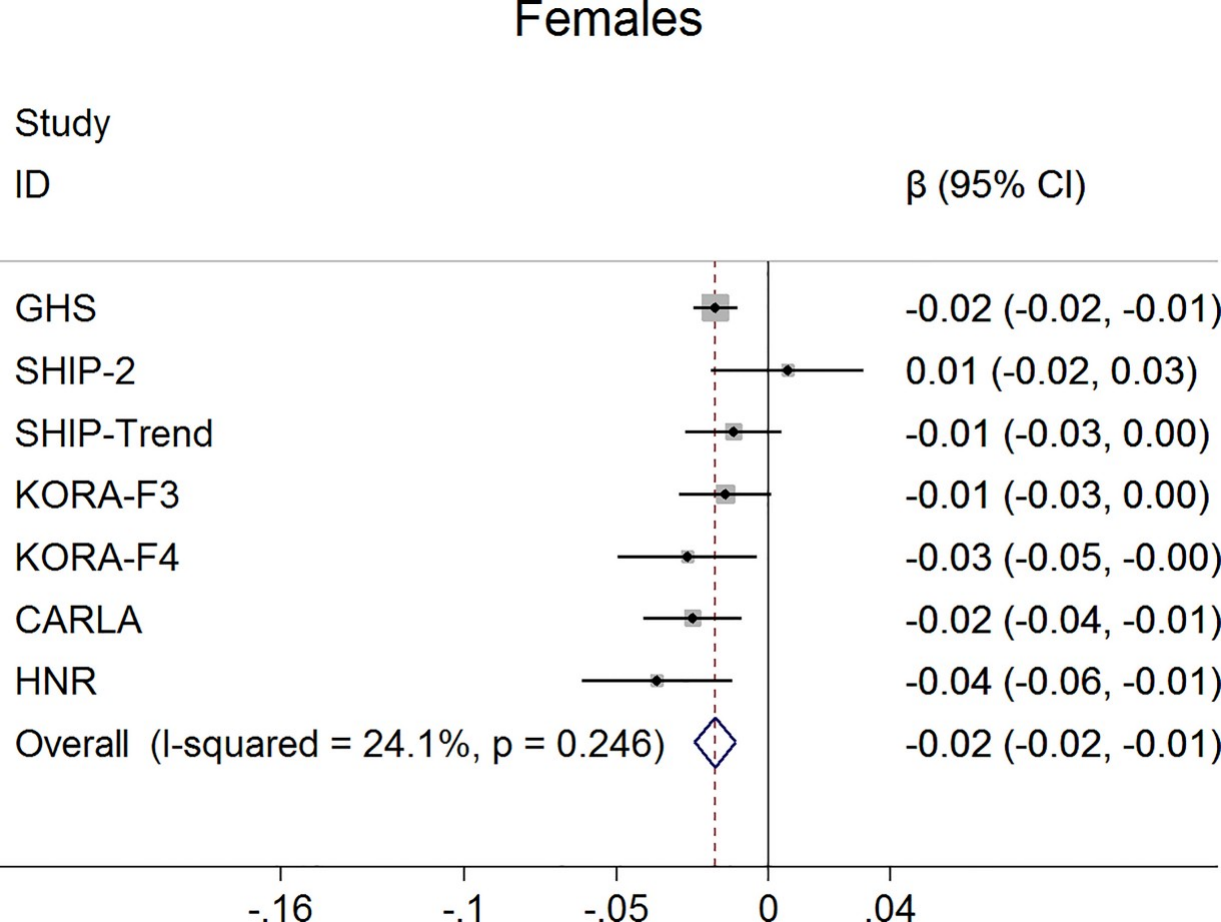
Association between claudication (definition 1) and ABI in women.

**Fig 3:**
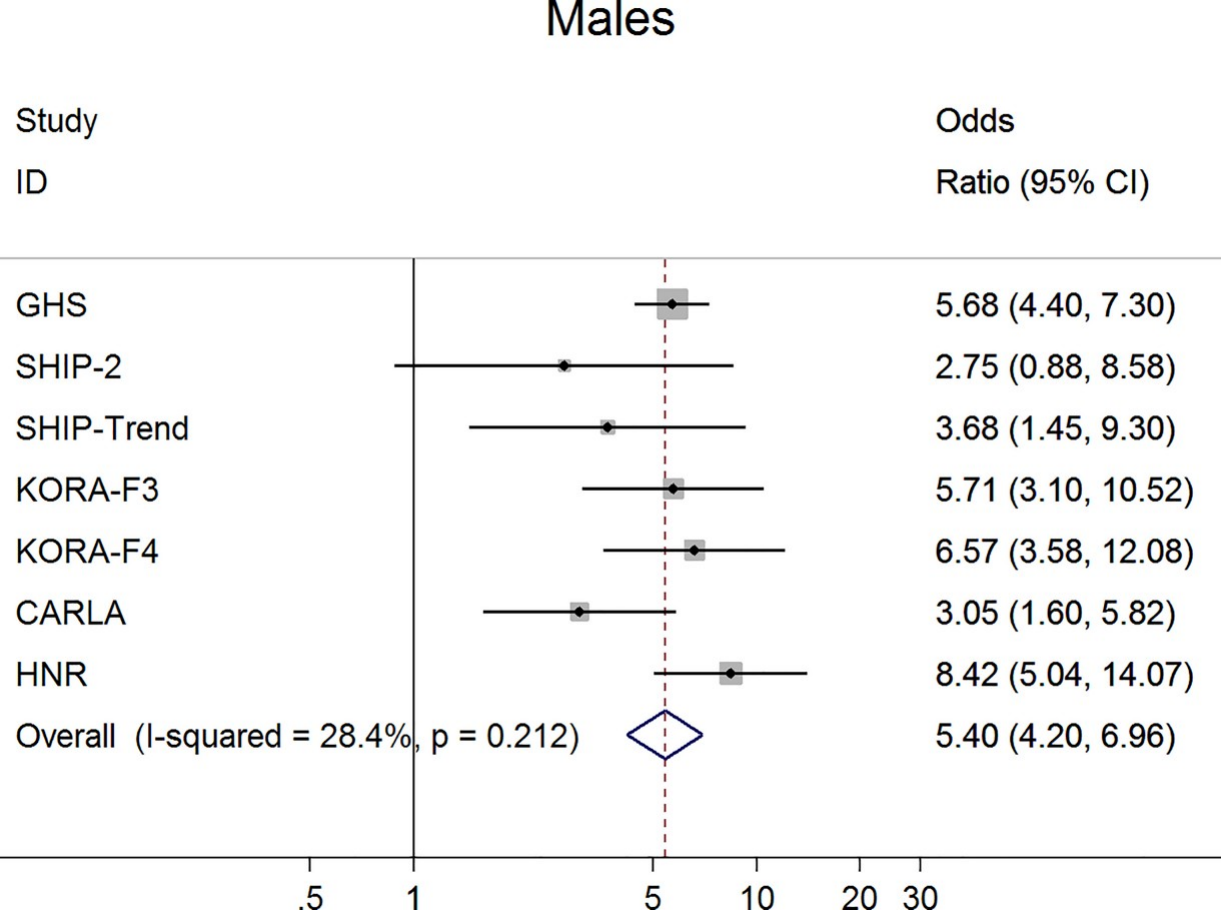
Association between claudication (definition 1) and low ABI in men.

**Fig 4:**
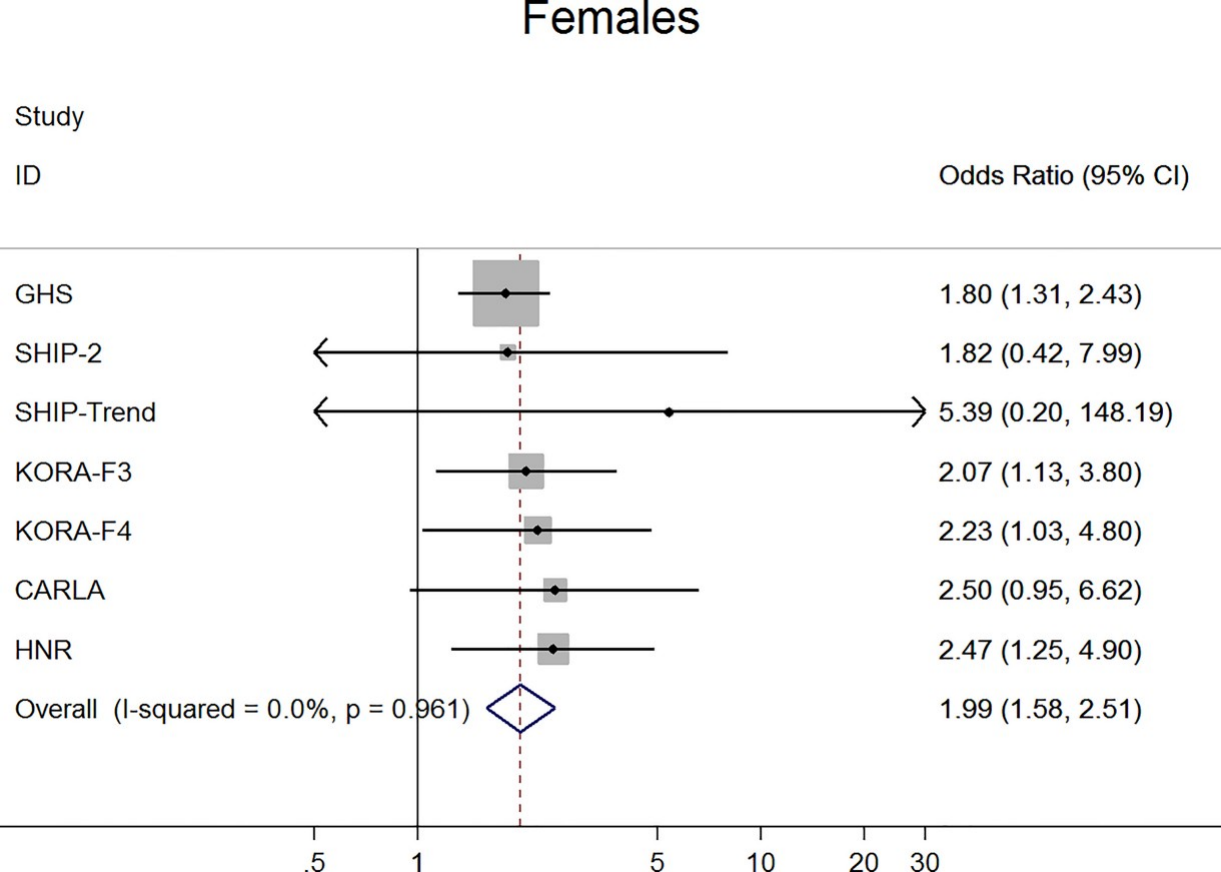
Association between claudication (definition 1) and low ABI in women.

For claudication definition 1, the meta-analysis demonstrated a significant negative association between claudication and ABI, which was more pronounced in men. Specifically, men with claudication had a 0.07 units lower ABI than those without claudication (95% confidence interval -0.10, -0.04; p<0.001) (Fig 1). This difference was markedly smaller in women (ß = -0.02; 95% confidence interval -0.02, -0.01; p<0.001) (Fig 2). Furthermore, in logistic regression analysis claudication was associated with a significantly higher probability for an ABI <0.90 and this association was stronger in men (odds ratio 5.4; 95% confidence interval 4.20, 6.96; p<0.001) (Fig 3) compared to women (odds ratio 1.99; 95% confidence interval 1.58, 2.51; p<0.001) (Fig 4).

For claudication definitions 2 and 3, effect sizes and Odds Ratios increase with increasing complexity of the definitions, both in men and women. For definition 3, which is only available in both KORA studies and GHS, though, Odds Ratio for low ABI is 13.99 (95% confidence interval 9.37, 20.89; p<0.001) in men and 8.16 (95% confidence interval 2.98, 22.32; p<0.001) in women.

### Sensitivity and specificity of claudication definitions

Calculation of sensitivity and specificity of each of the three definitions to detect low ABI, based on data from the largest cohort, GHS, revealed a low sensitivity for all definitions which decreases markedly from definition 1 to 3. The specificity is very high (>90%) for all claudication definitions with a slightly lower value for definition 1 as compared to definitions 2 and 3 (Table 3).

**Table 3:** Sensitivity and specifity of claudication definitions to detect low ABI in GHS.

Age group	35 to 74 years		55 to < 65 years		≥ 65 to 74 years	
Definition	Sensitivity	Specificity	Sensitivity	Specificity	Sensitivity	Specificity
**Definition 1**	23.6% [20.6; 26.8]	93.3% [92.9; 93.7]	28.5% [22.6; 35.1]	91.8% [90.8; 92.6]	32.6% [27.3; 38.2]	90.5% [89.5; 91.5]
**Definition 2**	11.8% [9.6; 14.3]	98.6% [98.4; 98.8]	15.0% [10.5; 20.4]	98.3% [97.9; 98.7]	16.1% [12.1; 20.8]	98.0% [97.5; 98.5]
**Definition 3**	9.8% [7.8; 12.2]	99.1% [98.9; 99.2]	12.6% [8.5; 17.8]	99.0% [98.6; 99.3]	13.4% [9.8; 17.8]	98.7% [98.2; 99.0]

## Discussion

### Improving PAD awareness

Focusing on cardiovascular and cerebrovascular event prevention, we need to identify early PAD stages in order to be able to reduce morbidity and mortality rates in these individuals. Our analysis demonstrates the advantages of a simple first step- screening strategy for PAD which markedly improves sensitivity accompanied by minor declines in specificity in comparison to a more complex questionnaire. Adopting this single question about pain or cramps in the legs during normal walking in history taking of general practitioners would help to achieve the guideline requirement to implement and support medical and public awareness of PAD[1]. This would certainly identify only a minority of individuals with PAD, but that is still a lot better than identifying none.

The narrow 95% confidence intervals overall and especially in the largest cohort (GHS) (Fig 1) demonstrate, that ABI differences were consistent between men with and without pain or cramps in the legs during normal walking.

### Patient workup

This single, simple question for claudication should serve as first screening step in all patients of a general practitioner who are at least 40 years old, as PAD prevalence already reaches 5% in women and men at age 45–49 years in high- income countries[3]. If individuals answer “yes” to this claudication question, the next step should be an ABI measurement. A primary ABI screening in the above mentioned patients would be preferable, as individuals with asymptomatic PAD could also be identified. However, despite falling costs of automated devices for ABI measurement, which enable less time-consuming and easier ABI screening, they are not yet widely available among general practitioners (also due to reimbursement issues), making a primary ABI assessment currently not feasible.

In diabetics, additionally an oscillography, a duplex ultrasound of the lower extremity arteries and/or toe-brachial index (TBI) measurements should be performed, as ABI measurements may not be correct in individuals with mediasclerosis of the leg arteries. In this analysis, we did not account for elevated resting ABI in diabetics. As we focus on detection of PAD at an early stage, we decided not to exclude subjects with known diabetes mellitus, because primary care physicians and patients may not know about diabetes mellitus at first presentation. It is reassuring to see significant results even with the inclusion of diabetics.

If PAD is diagnosed, patients should be treated according to current guidelines. This should include statin therapy[5] and a platelet aggregation inhibitor like aspirin or clopidogrel. Considering the results of the COMPASS PAD trial, patients with stable symptomatic PAD will probably soon receive a combination of aspirin and low-dose rivaroxaban[33].

If patients answer “no” to this claudication question, this does not exclude the presence of asymptomatic PAD. Further work-up for PAD should especially be considered in patients with diabetes, hypertension, hypercholesterolemia, positive family history of atherosclerotic disease, impaired renal function and smokers. It has been demonstrated, that even mild-to-moderate chronic kidney disease is associated with an increased risk of incident PAD[34]. Patients with known coronary artery disease usually do not need further work-up for asymptomatic PAD, as preventive measures will usually not change and interventional or surgical therapy of asymptomatic PAD is not indicated[1].

### Claudication definitions

Claudication- definition 1 is unspecific and may include “claudication” from non-ischemic causes. Claudication- definition 3 (Edinburgh Questionnaire) markedly underestimates PAD according to ABI <0.90—definition. While the Edinburgh Questionnaire was found to be highly sensitive in comparison to a subjective physician diagnosis of intermittent claudication, in this study it is not very sensitive in comparison to the generally accepted PAD-diagnosis based on ABI-measurement. There seems to be a relevant number of individuals, whose complaints do not meet the Edinburgh questionnaire criteria, but who are PAD-patients according to ABI-measurement.

It is important to keep in mind that most PAD patients are asymptomatic. But if we manage to motivate general physicians to ask their patients one question about claudication and thereby identify 20–30% of patients with mild PAD (Table 3), this will be a huge step forward in comparison to the present situation, in which the percentage of patients with mild PAD (still being a silent “killer”) who are recognized in general practices is probably close to 0%.

PAD-patients suffer from a bad prognosis. Therefore, early identification of these patients, which enables modification of risk factors and early pharmacological treatment, seems important. Consequentially, the higher sensitivity of an unspecific question seems more important than the slightly higher specificity of a complex questionnaire. In our opinion, it is about time to leave the traditional questionnaires in favor of one simple question. The data of our meta-analysis support to include this very simple one-item questionnaire into the daily clinical practice and into angiological and other vascular guidelines. Future claudication questionnaires should be evaluated in comparison with ABI measurements, not in comparison with the clinical evaluation by a physician. The analysis of the GHS data is in accordance with the results of the meta-analysis supporting the application of one question on claudication when screening for PAD.

### PAD symptoms in women

One question about claudication (definition 1) can identify individuals with a high likelihood of low ABI. The weaker association in women when compared to men could point to a different–so far unrecognized- clinical presentation of PAD in women. Already in 1982, a study from Finland demonstrated that the validity of “typical” claudication symptoms was poorer in women than in men and they were also less reliable predictors of death in women[15]. The Rose Questionnaire was evaluated in 55 patients, among them 37 with “undoubted intermittent claudication” (rather meaning undoubted PAD)[22]. Among these 37, only 5 were women, which makes it very likely that typical PAD symptoms in women besides claudication were not recognized. In coronary artery disease, the different clinical presentation in women in comparison to men remained unrecognized for decades. We need future studies looking for so far unrecognized PAD symptoms in women.

### PAD screening by ABI measurement

To our knowledge this is the largest analysis of the association between claudication and ABI in population-based studies. Our data demonstrate that even one simple question about claudication can help to identify individuals with PAD. However, these results do not negate the advantages of a PAD screening by ABI measurement. An ABI measurement allows to identify the large group of individuals with asymptomatic PAD[35], especially among older women. ABI measurements may be less time-consuming and cheaper than PAD prediction models which depend among others on laboratory values[36]. With the availability of simple and user-friendly-automated systems for the evaluation of ABI, PAD screening by ABI measurements has become easier. But as long as ABI screenings are not established in health care systems, asking one quick, simple question for claudication is clearly preferable to no PAD screening.

### Limitations

Some limitations have to be taken into account. The Edinburgh questionnaire for evaluation of claudication was not used in all studies of the present analysis. The number of participants varied widely among the studies. Rates of claudication and low ABI increase markedly at higher age, age groups were not homogenous in the seven population-based studies. The available data were not sufficient for calculation of the association between claudication and an alternative ABI calculation using the lowest leg pressure[37]. The perception of claudication may be influenced by exercise training [38], but this should be of lower importance in population-based studies.

## Conclusion

At least in men, one claudication question [asking for pain or cramps in the leg(s) during normal walking] can identify many individuals with a high likelihood of PAD and should be established as first screening step.

## Supporting information

S1 Data Data access information. (DOCX)
