# Supplementary material for: One simple claudication question as first step in Peripheral Arterial Disease (PAD) screening: A meta-analysis of the association with reduced Ankle Brachial Index (ABI) in 27,945 subjects
Source: PLoS One. 2019 Nov 4;14(11):e0224608. doi: 10.1371/journal.pone.0224608 (PMC6827909; doi:10.1371/journal.pone.0224608)
Supplement: S1 Data — (DOCX) [file pone.0224608.s001.docx]

**Data Availability Statement:**

Data of the cohort participants are subject to ethical and national data protection laws and are available only through an individual project agreement with the respective cohort. The authors had no special access privileges to the data. Phenotype data is available from all cohorts by request to the respective contact person’s or cohort steering committees for researchers who meet the criteria for access to confidential data. Information about how to access to the specific data for each cohort can be found in the Supporting Information.

Requests for data of the Cardiovascular disease, living and ageing in Halle (CARLA) study are subject to approval by the CARLA Steering Committee and should be sent via e-mail to Dr. Alexander Kluttig (alexander.kluttig@medizin.uni-halle.de) or to the CARLA Steering Committee (imebmi@medizin.uni-halle.de).

The Gutenberg Health Study (GHS) followed the recommendations of the Declaration of Helsinki and was requested by the local data protection officer and ethics committee of the Chamber of Physicians of Rhineland-Palatinate, Germany (reference no. 837.020.07). Written informed consent was obtained from all participants and does not approve public access to the data. Access to data at the local database in accordance with the ethics vote is offered upon request at any time. Requests are subject to approval and should be sent to the GHS Steering Committee via e-mail (info@ghs-mainz.de).

Requests for data of the Heinz-Nixdorf-Recall-Studie (HNR) should be sent to Prof. Dr. K-H. Jöckel, k-h.joeckel@uk-essen.de and are subject to approval by the HNR Steering Committee (http://www.medizin.uni-halle.de/index.php?id=1109).

Participant data for the KORA cohorts can be applied for through an individual project agreement with KORA. Other interested authors can access the data in the same way the authors used to access the data. Applications for access to the data sets can be

found at the following link: http://www. helmholtzmuenchen.de/en/kora-en/information-

for-scientists/participating-in-kora/utilization-ofkora-data/index.html.

Data on SHIP participants can be applied for at: https://www.fvcm.med.uni-greifswald.de/dd_service/data_use_intro.php. Those interested can access the data in the same manner as the authors.
